# A mathematical model of Marburg virus disease outbreaks and the potential role of vaccination in control

**DOI:** 10.1186/s12916-023-03108-x

**Published:** 2023-11-14

**Authors:** George Y. Qian, W. John Edmunds, Daniel G. Bausch, Thibaut Jombart

**Affiliations:** 1 Centre for Mathematical Modelling of Infectious Diseases, London School of Hygiene & Tropical Medicine, London, UK; 2 Department of Engineering Mathematics, University of Bristol, Bristol, UK; 3 FIND, Geneva, Switzerland; 4 Department of Disease Control, Faculty of Infectious and Tropical Diseases, London School of Hygiene & Tropical Medicine, London, UK; 5 MRC Centre for Global Infectious Disease Analysis, Department of Infectious Disease Epidemiology, School of Public Health, Imperial College London, London, UK

**Keywords:** Marburg, Marburgvirus, Filovirus, Vaccination, Zoonotic, Transmission, Modelling

## Abstract

**Background:**

Marburg virus disease is an acute haemorrhagic fever caused by Marburg virus. Marburg virus is zoonotic, maintained in nature in Egyptian fruit bats, with occasional spillover infections into humans and nonhuman primates. Although rare, sporadic cases and outbreaks occur in Africa, usually associated with exposure to bats in mines or caves, and sometimes with secondary human-to-human transmission. Outbreaks outside of Africa have also occurred due to importation of infected monkeys. Although all previous Marburg virus disease outbreaks have been brought under control without vaccination, there is nevertheless the potential for large outbreaks when implementation of public health measures is not possible or breaks down. Vaccines could thus be an important additional tool, and development of several candidate vaccines is under way.

**Methods:**

We developed a branching process model of Marburg virus transmission and investigated the potential effects of several prophylactic and reactive vaccination strategies in settings driven primarily by multiple spillover events as well as human-to-human transmission. Linelist data from the 15 outbreaks up until 2022, as well as an Approximate Bayesian Computational framework, were used to inform the model parameters.

**Results:**

Our results show a low basic reproduction number which varied across outbreaks, from 0.5 [95% CI 0.05–1.8] to 1.2 [95% CI 1.0–1.9] but a high case fatality ratio. Of six vaccination strategies explored, the two prophylactic strategies (mass and targeted vaccination of high-risk groups), as well as a combination of ring and targeted vaccination, were generally most effective, with a probability of potential outbreaks being terminated within 1 year of 0.90 (95% CI 0.90–0.91), 0.89 (95% CI 0.88–0.90), and 0.88 (95% CI 0.87–0.89) compared with 0.68 (0.67–0.69) for no vaccination, especially if the outbreak is driven by zoonotic spillovers and the vaccination campaign initiated as soon as possible after onset of the first case.

**Conclusions:**

Our study shows that various vaccination strategies can be effective in helping to control outbreaks of MVD, with the best approach varying with the particular epidemiologic circumstances of each outbreak.

**Supplementary Information:**

The online version contains supplementary material available at 10.1186/s12916-023-03108-x.

## Background

Marburg virus disease (MVD) is an acute haemorrhagic fever caused by Marburg virus (genus *Marburg marburgvirus*, family *Filoviridae*), affecting humans and nonhuman primates [1–4]. Marburg virus is zoonotic, maintained in nature in Egyptian fruit bats (*Rousettus aegyptiacus*), which are found across Africa [5]. Although rare, sporadic cases and outbreaks occur, usually associated with exposure in mines or caves inhabited by colonies of these bats [5–13]. Secondary human-to-human transmission may occur through direct exposure to blood, body fluids or contaminated surfaces.

**Fig. 1:**
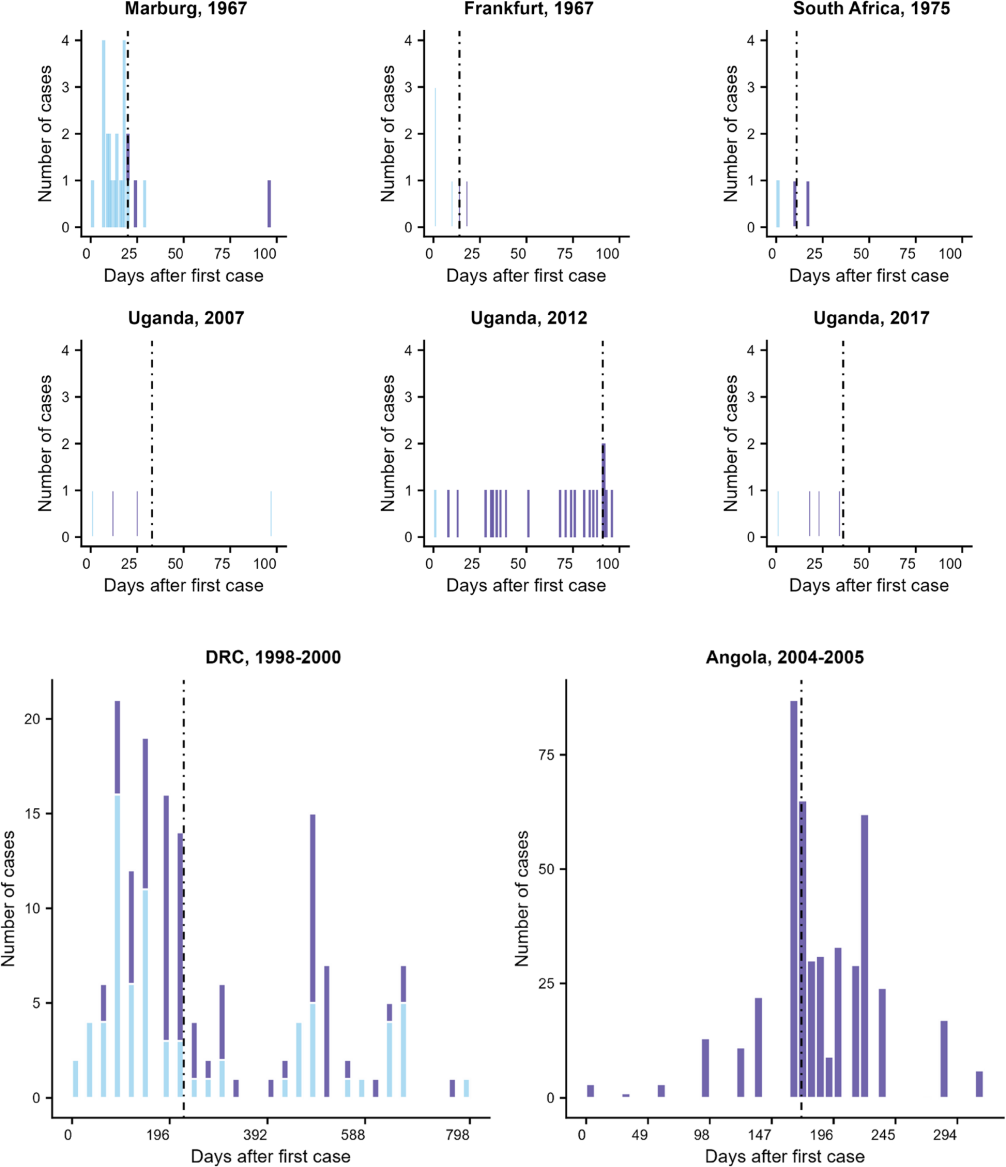
Incidence plots of Marburg virus disease outbreaks involving three or more confirmed or probable cases. Cases are plotted by onset date, except for the outbreak in Angola, which shows the day of reporting. Dashed lines represent the day on which interventions were put in place. Light blue bars indicate probable spillover infections to humans from animals and dark blue bars cases of human-to-human transmission. Spillover cases were usually explicitly reported in the literature, apart from the outbreak in DRC. For this outbreak, we assumed that miners who had been in contact with cases were secondary cases and those who were not were assumed to be infected from the reservoir. The other seven known outbreaks had fewer than 3 recorded cases

There have been 15 recognised MVD outbreaks up until 2022, beginning in 1967 when infected green monkeys from Uganda were imported to Germany and Yugoslavia for harvesting of their tissues for vaccine production [3, 14] (Additional file 1: Table S1). Excluding laboratory infections, the exposures of all index cases of outbreaks since then have occurred in Africa [5–13], with some outbreaks driven by recurring virus spillover from animals to humans and others primarily by human-to-human transmission. Figure 1 and Table S1 (in Additional file 1) show the MVD case numbers observed during each of the first fifteen outbreaks.

No licensed vaccine for MVD currently exists, although several are under development [4]. All previous outbreaks were controlled when transmission chains ended either naturally or through the introduction of public health and infection control measures [7, 15]. Nevertheless, the outbreak that occurred in Angola in 2004–2005, which registered 374 cases and 329 deaths (case fatality of 88 percent), illustrates the serious and explosive potential of Marburg virus. Furthermore, even in the smaller outbreaks, the high case fatality ratio could potentially be mitigated by vaccination [4].

The aims of this study were to estimate key epidemiological parameters of MVD, such as the reproduction number and serial interval, by collating data from the first 15 human outbreaks and subsequently use this information to parameterise a model that is used to assess the impact of different vaccination strategies to control outbreaks.

## Methods

### Data

We used linelist data from all except one of the first 15 outbreaks to estimate the serial interval of MVD. The one exception was the Angola outbreak that occurred during 2004–2005; here, because no linelist was available, we used case numbers reported periodically by the World Health Organization (in [16], for instance).

From the linelist data, we identified discernible infector-infectee pairs, obtained the difference between the dates of infection of each pair and fit appropriate distributions to these differences. We also used the linelist data to obtain the number of zoonotic introductions seen in each outbreak and, together with knowledge of the outbreak duration, estimated the rate of introductions.

We note that there are yet to be any confirmed cases of asymptomatic human MVD [16]. Moreover, the subsequent analysis assumes that there are no unobserved cases.

### Rate of zoonotic introductions

Several MVD outbreaks were driven primarily by zoonotic introductions, while others were largely caused by human-to-human transmission (Fig. 1). Thus, we estimated rates of introductions for two scenarios: one typical of a spillover-driven outbreak and one involving a single spillover but subsequently driven by human-to-human transmission. These two scenarios were typified by the outbreaks in the Democratic Republic of the Congo (DRC) [6] and Angola [17], respectively. We divided the number of spillover cases in each of these outbreaks by the outbreak duration to estimate a typical rate of introductions for each scenario.

The DRC outbreak spanned 2 years, with the first case being identified in October 1998 and the last in September 2000 [6]. This was an outbreak that was dominated by contact with bats [6]. Indeed, it was reported that only 27% of infected miners from this outbreak had contact with another infected individual [6], from which we infer that 73% of all infected miners were likely to be spillover cases. To estimate the rate of introductions, we divided the number of spillover cases by the outbreak duration [2 years]. This then constitutes an assumed typical rate of introductions during spillover-driven outbreaks.

Other outbreaks, such as the one that occurred in Angola [17], likely involved a single spillover event subsequently driven by human-to-human transmission. To obtain a typical rate of introductions for these, we divided the number of spillover cases by the duration of the Angola outbreak, during which it is believed that only one introduction occurred [17].

Since there have been only 15 recorded outbreaks from 1967 up until 2021, the total number of introductions across this time period must have been very low, even accounting for undetected outbreaks. To reflect this, we performed a sensitivity analysis where we lowered both the spillover- and transmission-driven rates of introductions, described above, by one and then two orders of magnitude.

### Time from first case to interventions

For each outbreak, we estimated the date on which interventions were put in place [3, 6–12, 18–22]. During earlier outbreaks, when little was known about MVD, this was either when the disease was acknowledged as being dangerous and highly transmissible (namely during the 1967 European outbreaks), or the date at which patients who showed symptoms consistent with other viral haemorrhagic fevers were identified and treated accordingly. This then prompted changes in clinical, laboratory and infection prevention and control practices [14], for instance through application of case isolation and barrier nursing [18]. Barrier nursing involves placing the individual in a separate area and healthcare workers taking extra precautions, ranging from wearing personal protective equipment, including head covering and eye protection, to disinfecting all objects in the area and showering immediately after going off-duty.

For later outbreaks, we used the day on which response teams were deployed to the region of the outbreak as the intervention date (while recognising that local control efforts were often already underway). We calculated the median time delay between onset of the first case and the beginning of interventions across all outbreaks. This median was used in forward simulations as the time between disease onset of the first case to the date when interventions were implemented. However, intervention during several outbreaks, including those in Angola (2004–2005) [19, 23], DRC (1998–2000) [6] and Uganda (2012) [20] took place several weeks after the median. Hence, as a sensitivity analysis, we also took the 75th percentile of this delay to intervention and modelled this scenario.

### Factors affecting outbreak size

The number of cases in each MVD outbreak is presumed to be dependent on several factors, including the number of zoonotic introductions, delay from first case to intervention and calendar year in which the outbreak occurred. The impact of armed conflict was also noted as a possible factor for the two largest MVD outbreaks—DRC and Angola [6, 18, 24]—though we note that conflict in both these regions had officially ended shortly before the outbreaks occurred.

We used a negative binomial generalised linear model (GLM) to test if the total number of secondary cases (i.e. excluding zoonotic introductions) was linked with these four potential covariates (initial model). We also investigated a reduced model which omitted the covariables that were not significantly associated with the total number of secondary cases (*p*>0.05). A negative binomial distribution for the response variable was preferred to a Poisson because of the high over-dispersion that may be associated with this variable—as is in the case of Ebolavirus [25]. Note that such dispersion was not observed in daily case incidence within individual outbreaks, which we later on modelled using a Poisson branching process.

### Branching process model

We used a branching process to model MVD transmission over time. New infections generated at any time, *t*, are governed by the force of infection *λ*_*t*_, which is determined by previous case incidence *y*_*s*_ (*s* = 1, …, *t*−1), the serial interval distribution (denoted by *w*, its probability mass function), and the reproduction numbers *R*_*s*_ as:

1 $$\lambda_t = \sum_{s=1, \dots,\,t-1}\,\,R_s\,y_s\,w(t - s)$$

There are multiple ways to estimate the reproduction number from case data [26–28]. Here, we are interested in the case reproduction number [26], estimated using Eq. 1, rather than the instantaneous reproduction number [27], since this is a retrospective analysis. Moreover, we have information on serial intervals, through dates of symptom onset, but not dates of infection and so the former is also more appropriate [28]. Though the case reproduction number gives estimates that are shifted forward in time [28], in contrast to the instantaneous number, the analysis we have conducted is retrospective, rather than in real time.

We assume that higher-risk groups are at higher risk of exposure, either to the reservoir, as in the case of miners, or to cases by means of being a healthcare worker, but not higher risk of transmission to others. Hence, assuming the same average reproduction number for all individuals, new secondary cases at time *t* are then drawn from a Poisson distribution so that:

2 $$y_t \sim\text{ Poisson}(\lambda_t)$$

Equation (1) shows that the reproduction number *R*_*s*_ is allowed to vary over time. This is used to distinguish, in any given outbreak, two phases: the first during which transmission is maximum (*R*_*s*_ = *R*_0_, the basic reproduction number), and the second during which intervention reduces transmission by a factor *E*, the intervention efficacy, so that:

3 $$R_s = R_0 (1 - E)$$

Intervention is defined, in this context, as the implementation of measures such as case isolation, contact tracing and barrier nursing. We assume that depletion of the susceptible population is negligibly low, given the low number of cases compared to the population of each affected community.

In our model, the reduction in the reproduction number from *R*_*0*_ to *Rs* assumed to occur instantaneously after the date after which interventions are implemented (see subsection above).

The model also incorporates a constant rate of introductions, *γ*, in which newly introduced cases (presumed spillover events) are also Poisson distributed. Hence, the number of new cases at time $t$, ${Y}_{t},$ is the sum of the primary and secondary cases:

4 $${Y}_{t} \sim \mathrm{ Poisson}\left(\gamma \right)+\mathrm{Poisson}({\uplambda }_{t})$$

### Parameter estimation

We used an Approximate Bayesian Computational (ABC) framework for estimating the basic reproduction number, *R0*, and *E* for each outbreak separately. To do this, we first determined the delay to implementation of interventions, the duration of the outbreak and rate of introductions for each particular outbreak. The priors used were as follows: *R*_*0*_ ∈ U(0,3) and* E* ∈ U(0,1). The summary statistic used was the absolute difference between the total number of cases observed in a simulation and the actual number reported during the outbreak. Parameter values were retained as part of the posterior sample if this difference was within 10% of the actual value:

$$\left|{n}_{simulated}-{n}_{outbreak}\right|<0.1 {n}_{outbreak}$$.

Five thousand posterior samples were retained in this way per outbreak.

For estimates of the serial interval, 26 infector-infectee pairs were determined from literature on previous MVD outbreaks. These infector-infectee pairs were determined by local epidemiologists in the field at the time investigating the outbreaks. Most of these were household transmission pairs: for instance, miners infecting a family member in the DRC outbreak [6]. Occasionally, however, the pair involved nosocomial transmission, as happened in the outbreak in Kenya [12]. Dates of onset for each pair, specified by epidemiologists, were used to estimate the serial interval by fitting four distributions (gamma, negative binomial, Poisson and logistic) to the time period between the dates of onset for these infector-infectee pairs using the *fitdistrplus* package in *R*. The Akaike Information Criterion (AIC) was used to determine the best-fit model.

We then modified the model to include the effects of vaccination on transmission. Vaccination reduces the reproduction number associated with each case by the vaccine efficacy (*VE*) corresponding to that case, on top of any reduction due to intervention efficacy I. Six vaccination schemes were simulated:

1. *Prophylactic targeted vaccination of high-risk groups*. This scheme involves the vaccination of healthcare workers, as well as individuals who reside near or work in mines or caves. From a search of linelist data [3, 6–14, 17, 18, 20, 21, 23], we estimate that, across all MVD outbreaks, approximately 6% of cases were healthcare workers and 12% were individuals living near or working in mines or caves (though this excludes the outbreak in Angola due to lack of data).
2. *Prophylactic mass vaccination*. The second scheme was prophylactic mass vaccination of the entire community prior to the outbreak
3. *Ring vaccination*. This is the first of the reactive vaccination strategies that we simulated. A proportion of contacts are vaccinated after the date of intervention. This proportion depends on how extensively case reporting is carried out, as well as on vaccination coverage
4. *Reactive targeted vaccination*. This scheme entails vaccination of the same high-risk groups as in the prophylactic targeted vaccination, but done reactively after an MVD outbreak has begun. In our model, vaccination is simulated only after the date of intervention
5. *Reactive mass vaccination*. This is mass vaccination simulated only after intervention has begun
6. *A combination of ring and reactive targeted vaccination schemes*

For all vaccination schemes, we assumed that no waning of immunity occurred after vaccination. The vaccination parameters in our model were:

Maximum Vaccine Efficacy $\left(VE_{max}\right)$:

$V{E}_{max}$ of a vesicular stomatitis virus (VSV)-based vaccine expressing the MARV glycoprotein (VSV-MARV) was found to be 100% in nonhuman primates (NHPs) [2]. As this is unlikely to be observed in the field, we adjusted downward to a $V{E}_{\mathrm{max}}$ of 90% in the base case.

Time from vaccination to maximum vaccine efficacy:

We assumed that the time from vaccination, when efficacy is 0%, to maximum efficacy was 7 days. This was the time period observed during Phase I trials on NHPs of the MVD vaccine [2]. In our model, we represented the increasing vaccination efficacy over time through a logistic curve (Additional file 1: Figure S1).

### Vaccination against transmission

We have assumed that a potential MVD vaccine reduces onward transmission and, therefore, disease (though not disease in the cases themselves). However, we also relax this assumption by conducting a sensitivity analysis where the vaccine prevents disease but not transmission.

### Vaccine coverage

The level of coverage that is likely to be achieved in an outbreak setting is difficult to estimate. In two reviews of oral cholera vaccine, uptake coverages ranged between 61 and 100% for at least 1 dose and 46 and 92% for two dose coverage in outbreak settings. Hence in this analysis, in the base-case, we assume that targeted and ring vaccination coverages would be 70%, at the lower end of the one-dose coverage, since MVD vaccines currently in the pipeline are injectable, rather than oral (as is the case with oral cholera vaccines).

Given logistical difficulties and possible vaccine shortages [29], we would expect a lower rate of coverage (assumed to be 50%) in the case of a mass vaccination strategy—both reactive and prophylactic. We increased or decreased these coverages by 20% in the sensitivity analyses.

### Conditional timing between vaccination and infectious contact

The time between vaccination and effective contact with an infectious person or animal is not independent in a reactive programme. This is because contacts of cases (in the case of ring vaccination) or the wider community (mass vaccination) are vaccinated in response to a case. Thus, many people will be vaccinated around the time that they are infected, lowering vaccine efficacy. We have taken this into account by assuming that, amongst individuals who become infected, the average delay between vaccination and infectious contact was normally distributed with a mean of 20 days (s.d. 5 days) for prophylactic and 9 days (s.d. 4 days) for reactive strategies. These normal distributions (truncated at zero days) were chosen so that vaccination efficacy varied during reactive vaccination, with most of the efficacy values lying on the slope of the logistic curve fit of vaccine efficacy (see next section), while for prophylactic vaccination, over 99% of values were within 0.5% of the maximum vaccine efficacy.

Data from a previous ring vaccination trial for an Ebola virus disease vaccine in Guinea showed an average delay from vaccination to subsequent infection (given the individuals had indeed been infected) in the rings of 5.7 days (s.d. 5.0 days) [30]. We also examined this delay distribution as a sensitivity analysis.

### Logistic curve fit of vaccine efficacy

We modelled the vaccine efficacy from vaccination to infectious contact using a logistic curve of the form:

$$VE\left(t\right)=\frac{V{E}_{max}}{1+\mathrm{exp}\left(\frac{{t}_{mid}-t}{scale}\right)}$$

where $t$ is the number of days after vaccination, $V{E}_{\mathrm{max}}$ is the maximum vaccine efficacy, ${t}_{\mathrm{mid}}$ is the day when $VE$ reaches its inflection point and $scale$ is the scaling parameter on the *x*-axis (Figure S1 in Additional file 1). A nonlinear least squares approach (specifically, the Levenberg-Marquardt algorithm) was used to fit this curve so that $VE$ increases from 0 to within 0.5% of the maximum $VE$, denoted $V{E}_{\mathrm{max },}$ in 12 days (see [2]) and subsequently tends towards this maximum.

### Forward simulations

We subsequently performed forward simulations to show the effects of these different vaccination schemes on potential outbreaks under both the low and high introduction rates estimated previously. For each forward simulation, we selected at random one set of parameters from the posterior distribution of one randomly selected MVD outbreak. We ran 10,000 simulations per vaccination scheme in this manner. The maximum number of cases for each outbreak was restricted to 5000.

We compared the distributions of simulated case numbers after implementing the six vaccination strategies described above with the no-vaccination scheme. We also estimated the proportion of terminated outbreaks predicted under each scheme. An outbreak was considered to be terminated if the force of infection (see Eq. 2) was less than 0.05 after 365 days. Such a rate would be equivalent to less than one new infection per 20 days, which is at the upper limit of the incubation period for MVD [1]. This would correspond to an outbreak that has effectively ended after 1 year.

## Results

### Epidemiological parameters

The estimated rate of zoonotic introductions was 0.06 and 0.003 per day during the DRC and Angola outbreaks, respectively. Across all outbreaks, the median delay between onset of the first MVD case and beginning of interventions was 21 days.

A gamma distribution was found to best fit the MVD serial intervals, according to the AIC (Additional file 1: Table S2). The fitted gamma distribution of the serial intervals had a mean of 9.2 days and standard deviation of 4.4 days (Additional file 1: Figure S2). The median value of ${R}_{0}$ was 0.8 [95% CI 0·08–1·8], while the median value of ${R}_{s}$, the post-intervention reproduction number, was 0.3 [95% CI 0·01–1·3], from Eq. 3. These values should, however, be interpreted with caution due to the heterogeneity of the reproduction number across all MVD outbreaks.

**Fig. 2:**
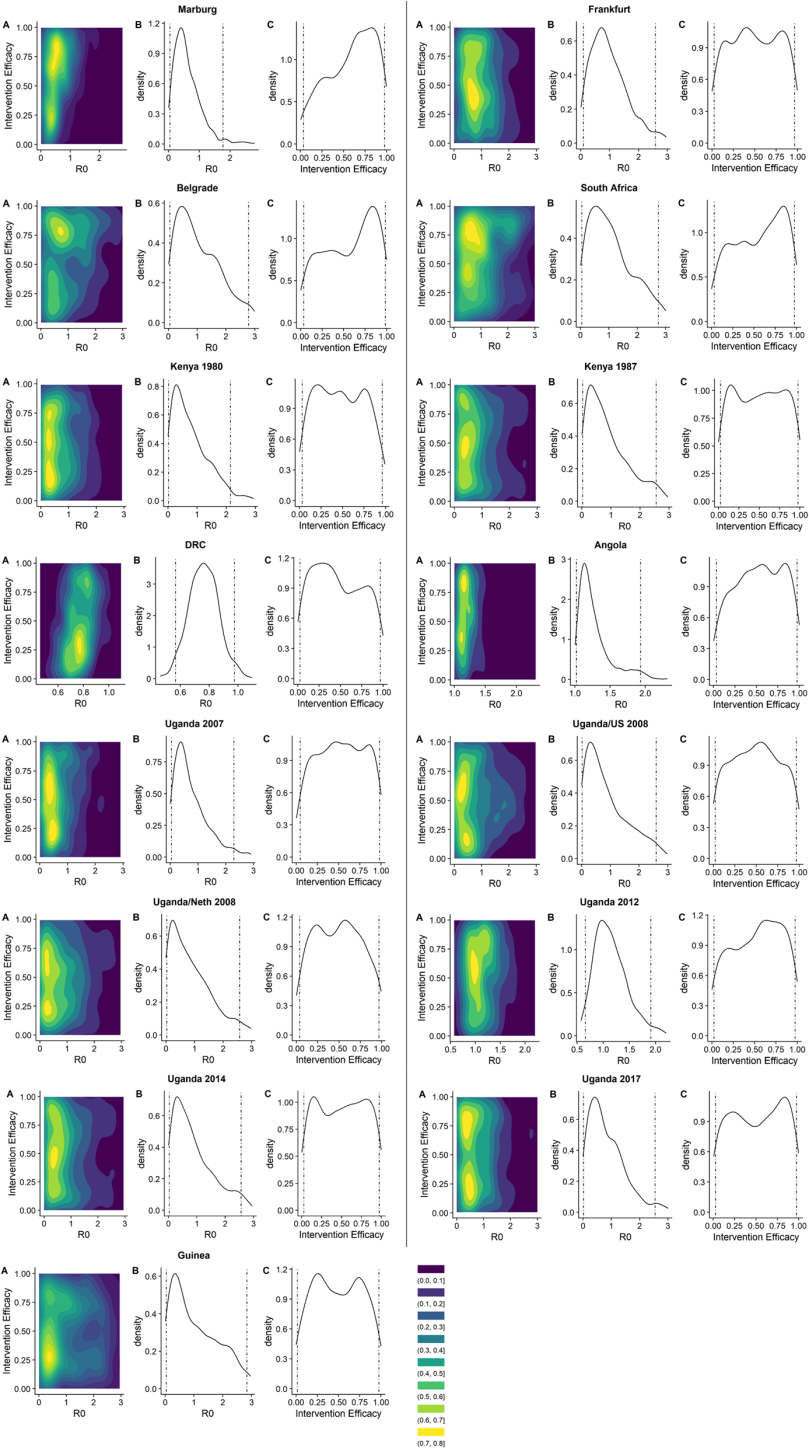
**A** Posterior distribution of the basic reproduction number and intervention efficacy combined. **B** Marginal posterior distribution of the basic reproduction number. **C** Marginal posterior distribution of the intervention efficacy for each outbreak, in chronological order. Dashed lines indicate 95% confidence intervals. Purple/blue regions in plot A represent low density pairs of intervention efficacy and ${R}_{0}$ values (i.e. pairs with relatively few values found in the posterior distribution), while yellow regions represent high density pairs (i.e. pairs with relatively many values found in the posterior distribution)

Instead, it is more informative to inspect the distributions of ${R}_{0}$ and the post-intervention reproduction number for each previous outbreak: these are shown in Fig. 2. The value of ${R}_{0}$ for each individual outbreak ranged from 0.5 [95% CI 0.05–1.8] to 1.2 [95% CI 1.0–1.9], while the post-intervention reproduction number ranged from 0.2 [95% CI 0.006–0.7] to 0.6 [95% CI 0.03–1.5]. For the majority of outbreaks, the median value of ${R}_{0}$ was less than 1, indicating there was little person-to-person transmission even before interventions. Consequently, the intervention efficacy is difficult to estimate during these outbreaks. There were, however, two outbreaks where ${R}_{0}$ was likely to have been greater than 1: namely the Angolan outbreak in 2004–2005 and Ugandan outbreak in 2012, where ${R}_{0}$ was estimated to be 1.2 (95% CI 1.0–1.9) and 1.1 (95% CI 0.7–1.9) respectively. For these outbreaks, our estimates of the intervention efficacy were skewed towards higher values (Fig. 2). These parameters are summarised in Table 1.

**Table 1:** Epidemiological parameters associated with Marburg virus and Ebola virus diseases

**Parameter**	**Marburg virus disease**	**Ebola virus disease**
**Case fatality ratio**	53.8% [95% CI 26.5–80%] [31]	65.0% [95% CI (54.0–76.0%)] (All Outbreaks) [31]
**Serial Interval (Gamma distribution)**	Mean: 9.2 days Standard Deviation: 4.4 days^a^	Mean: 11.6 days Standard Deviation: 6.3 days^d^ [32]
**Incubation period (range of central values, (range))**	5–10 (2–21) days (15,31)	5.3–12.7 (1–21) days^d^ [1, 4]
**Basic reproduction number**	1.2 [95% CI 1.0–1.9] (Angola, 2004–2005) 0.76 [95% CI 0.57–0.98] (DRC, 1998–2000) 1.1 [95% CI 0.66–1.9] (Uganda, 2012) Median estimates range from 0.51 to 1.2, depending on outbreak^b^	1.71 [95% CI 1.44, 2.01] (Guinea) 1.83 [95% CI 1.72, 1.94] (Liberia) 2.02 [95% CI 1.79, 2.26] (Sierra Leone)^d^ [32]
**Maximum vaccine efficacy**	100% (on Nonhuman Primates)^c^ [2]	100% [30]
**Days between vaccination and maximum efficacy**	7 (on Nonhuman Primates)^c^ [2]	10 [30]
**Conditional timing between vaccination and infective contact**	7 (on Nonhuman Primates)^c^ [2]	Mean: 5.73 days Standard deviation: 5.03 days [30]
**Delay between onset of first case and implementation of interventions**	21 days (median over all outbreaks)^c^	~ 90 days^d^

^a^ Inferred from data

^b^ Simulated values

^c^ Assumed values, from Phase I trials of an MVD vaccine

^d^ Ebola virus disease outbreak in West Africa, beginning in 2013

### Factors influencing outbreak size

The negative binomial regression suggested that, of all factors that we investigated, there was evidence (*p*<0.001) that the length of delay to interventions influenced the number of secondary cases, but no evidence (*p*>0.05) that the other factors modelled did so (Table 1). Specifically, an increase of one day to this delay resulted in the log number of secondary cases increasing by a factor of 1.03 (the incidence rate ratio) (Table 1). The coefficient associated with the delay to intervention was very similar under the reduced model, which had this delay until interventions as its lone covariate (Table 2).

**Table 2:** Values of coefficients for the negative binomial regression (with a natural log link), for a model with the following covariates: number of zoonotic introductions, delay to interventions, calendar year of outbreak and whether armed conflict occurred shortly before

**Covariates**	**Estimated value**	**Standard error**	**Lower CI (2.5%)**	**Upper CI (97.5%)**	***p*-value**
**Model intercept**	41	44	−46	130	0.36
**Number of introductions**	−0.036	0.033	−0.10	0.029	0.27
**Delay to interventions**	0.028	0.0080	0.012	0.043	<0.001
**Calendar year of outbreak**	−0.020	0.022	−0.064	0.023	0.36
**Armed conflict**	1.3	1.5	−1.7	4.3	0.41

### Simulations of vaccination strategies

**Fig. 3:**
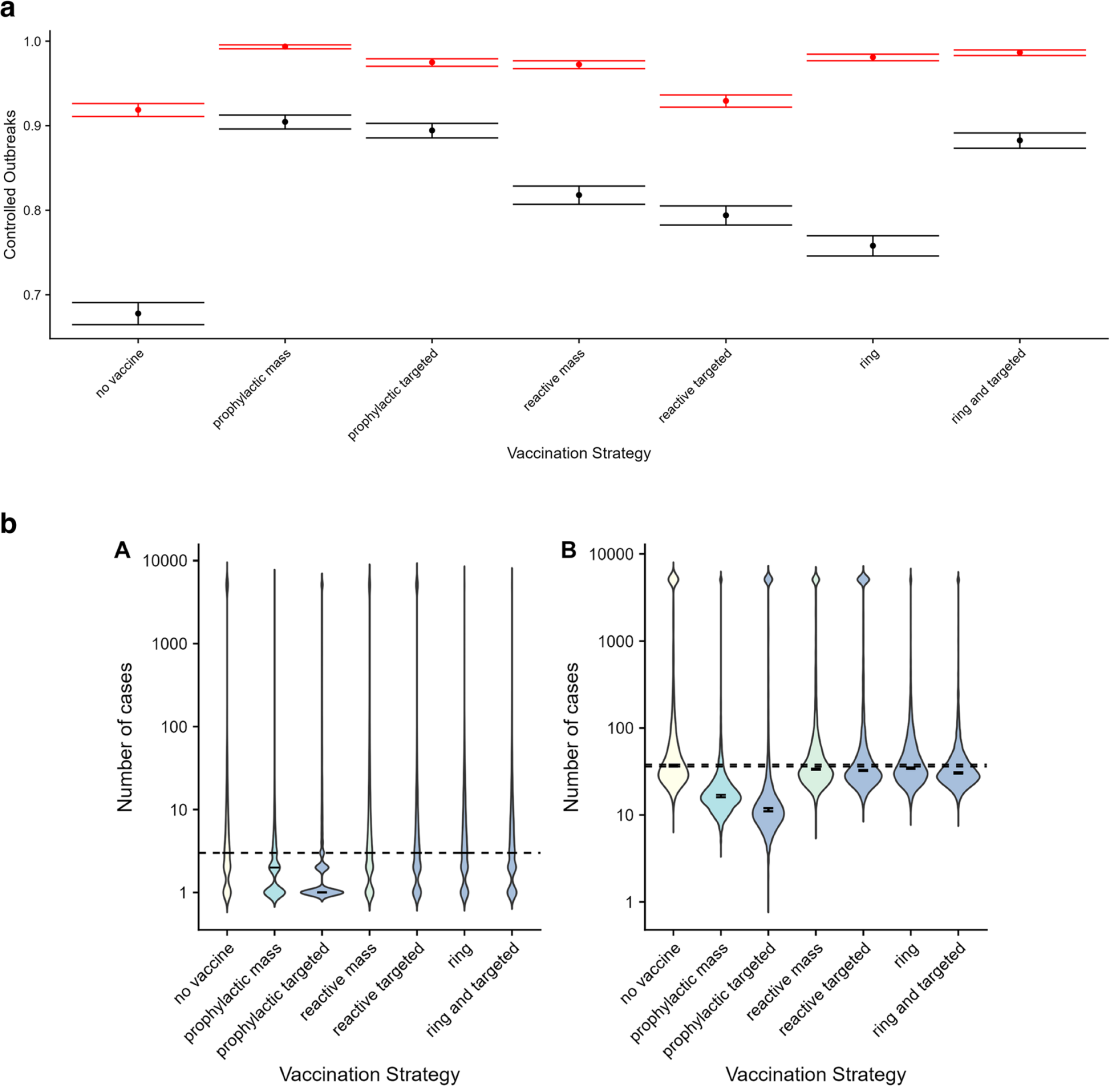
**a** Proportion of outbreaks terminated after 1 year, predicted under different vaccination strategies, when the rate of zoonotic introductions is low (red bars) and high (black bars). The error bars represent the upper and lower bounds of the 95% confidence intervals for each vaccination strategy. **b** Median number of cases per outbreak predicted under different vaccination strategies, when the rate of zoonotic introductions is low (panel A) and high (panel B). The dashed lines represent the upper and lower bounds of the 95% confidence intervals in the absence of vaccination (“no vaccine”)

The proportion of terminated outbreaks in the absence of any vaccination strategy was 0.92 (95% CI 0.91–0.93) and 0.68 (CI 0.67–0.69) when the rate of introductions was low and high, respectively. Most vaccination strategies resulted in an increase in this proportion; in particular the combined ring and targeted strategy, with values of 0.99 (CI 0.99–0.99) and 0.88 (CI 0.87–0.89), the prophylactic mass strategy, with values of 0.99 (CI 0.99–0.99) and 0.90 (CI 0.90–0.91), for low and high rates of introductions, respectively, and prophylactic targeted, with values of 0.98 (CI 0.97–0.99) and 0.89 (CI 0.88–0.90), for low and high rates of introductions, respectively (Table 3 and Fig. 3a).

**Table 3:** Values of coefficients for the negative binomial regression (with a natural log link), for a reduced regression model with the delay to interventions as the lone covariate. The null and residual deviances are 8 and 72, respectively and so the pseudo-${R}^{2}$ value is 0.89

**Covariates**	**Estimated value**	**Standard error**	**Lower CI (2.5%)**	**Upper CI (97.5%)**	***p*-value**
**Model intercept**	0.24	0.40	−0.60	1.1	0.55
**Delay to interventions**	0.030	0.0036	0.022	0.038	<0.001

The median number of cases in the absence of any vaccination strategy was 3 (95% CI 3–3) and 36 (CI 35–37) for low and high rates of introductions, respectively. Under the low rate of introductions scenario, there was a small decrease in this median to 2 cases for the prophylactic mass vaccination strategy, and to 1 case for the prophylactic targeted strategies, while there was also a small decrease observed for all reactive vaccination strategies when simulating a high rate of introductions, with the exception of the ring vaccination scheme. Under this higher rate of introductions, a larger reduction in the median number of cases under the prophylactic mass and targeted strategies was observed (17 and 11 cases, respectively) (Table 4 and Fig. 3b). We note that the boluses around the 5000 cases mark in Fig. 3b represent outbreaks that are both ongoing after 1 year (i.e. had a force of infection greater than 0.05 per day after 365 days) and had accumulated over 5000 cases (the maximum number of cases to which we had capped each simulated outbreak).

**Table 4:** Proportion of terminated outbreaks for different vaccination schemes. The numbers in parentheses indicate the 95% CIs of this proportion, from bootstrapped sampling

**Scheme**	**No vaccination**	**Prophylactic mass**	**Prophylactic targeted**	**Reactive mass**	**Reactive targeted**	**Ring**	**Ring and reactive targeted**
**Baseline (Low/High rate of introductions)**	0.92 (0.91–0.93)	0.99 (0.99–0.99)	0.98 (0.97–0.98)	0.97 (0.97–0.98)	0.93 (0.92–0.94)	0.98 (0.98–0.98)	0.99 (0.98–0.99)
	0.68 (0.67–0.69)	0.90 (0.90–0.91)	0.89 (0.88–0.90)	0.82 (0.81–0.83)	0.79 (0.78–0.81)	0.76 (0.75–0.77)	0.88 (0.87–0.89)
**Reduced time from reactive vaccination to infective contact (Low/High rate of introductions)**	0.92 (0.91–0.93)	0.99 (0.98–0.99)	0.98 (0.97–0.98)	0.96 (0.95–0.97)	0.93 (0.92–0.93)	0.94 ( 0.93–0.94)	0.97 (0.97–0.98)
	0.68 (0.67–0.69)	0.90 (0.90–0.91)	0.89 (0.88–0.90)	0.77 (0.76–0.78)	0.76 (0.75–0.76)	0.74 (0.73–0.75)	0.81 (0.81–0.82)
**Lower vaccination coverage (Low/High rate of introductions)**	0.92 (0.91–0.93)	0.97 (0.97–0.98)	0.96 (0.95–0.96)	0.95 (0.95–0.96)	0.94 (0.93–0.94)	0.96 (0.95–0.96)	0.97 (0.97–0.98)
	0.68 (0.67–0.69)	0.82 (0.81–0.83)	0.84 (0.83–0.85)	0.77 (0.76–0.78)	0.75 (0.73–0.76)	0.75 (0.74–0.76)	0.81 (0.80–0.82)
**Higher vaccination coverage (Low/High rate of introductions)**	0.92 (0.91–0.93)	1.00 (1.00–1.00)	0.99 (0.97–1.00)	0.99 (0.98–0.99)	0.93 (0.93–0.94)	0.98 (0.98–0.98)	1.00 (1.00–1.00)
	0.68 (0.67–0.69)	0.97 (0.97–0.98)	0.95 (0.95–0.96)	0.89 (0.88–0.90)	0.82 (0.81–0.83)	0.76 (0.75–0.77)	0.93 (0.93–0.94)
**Later date of intervention (Low/High rate of introductions)**	0.88 (0.86–0.88)	0.99 (0.99–0.99)	0.96 (0.95–0.96)	0.91 (0.90–0.92)	0.88 (0.87–0.89)	0.91 (0.90–0.92)	0.92 (0.91–0.92)
	0.64 (0.63–0.65)	0.91 (0.90–0.91)	0.87 (0.86–0.88)	0.78 (0.77–0.79)	0.75 (0.74–0.76)	0.71 (0.70–0.73)	0.82 (0.81–0.83)
**Vaccine protects against disease only (Low/High rate of introductions)**	0.92 (0.91–0.93)	0.92 (0.91–0.93)	0.92 (0.91–0.93)	0.92 (0.91–0.93)	0.92 (0.91–0.93)	0.92 (0.91–0.93)	0.92 (0.91–0.93)
	0.68 (0.67–0.69)	0.68 (0.67–0.69)	0.68 (0.67–0.69)	0.68 (0.67–0.69)	0.68 (0.67–0.69)	0.68 (0.67–0.69)	0.68 (0.67–0.69)
**Reduced introduction rate (by 1 order of magnitude)**	0.93 (0.93–0.94)	1.00 (0.99–1.00)	0.98 (0.98–0.99)	0.98 (0.98–0.99)	0.94 (0.93–0.95)	0.99 (0.99–0.99)	0.99 (0.99–0.99)
	0.88 (0.87–0.89)	0.98 (0.98–0.99)	0.96 (0.96–0.97)	0.96 (0.96–0.97)	0.92 (0.92–0.93))	0.96 (0.96–0.97)	0.98 (0.97–0.98)
**Reduced introduction rate (by 2 orders of magnitude)**	0.93 (0.93–0.94)	1.00 (0.99–1.00)	0.98 (0.98–0.99)	0.98 (0.98–0.99)	0.94 (0.93–0.95)	0.99 (0.99–0.99)	0.99 (0.99–0.99)
	0.93 (0.93–0.94)	1.00 (0.99–1.00)	0.98 (0.98–0.99)	0.98 (0.97–0.98)	0.94 (0.93–0.94)	0.99 (0.99–0.99)	0.99 (0.99–0.99)

### Sensitivity analyses

Varying the vaccination parameters had the following effects:

Reducing the conditional timing between vaccination and infectious contact to 5.73 days (s.d. 5.03 days) did not result in meaningful differences from baseline values in either the proportion of terminated outbreaks or the median number of cases across all simulated outbreaks (Tables 3 and 4).

Reducing vaccination coverage to 20% less than baseline values led to higher median number of cases than baseline, as well as a decrease in the proportion of terminated outbreaks across all vaccination schemes, apart from the ring (Tables 3 and 4). However, this was only apparent if the rate of introductions was high. With increased coverage (20% greater than baseline values), all vaccination strategies performed better than baseline in terms of increasing the proportion of terminated outbreaks, though no change in the median number of cases was observed (Tables 3 and 4).

When the date of intervention was increased to 90 days after onset of the first case, we observed a decrease in proportion of terminated outbreaks for reactive vaccination strategies at the higher introduction rate (Table 3). For instance, whereas 99% of outbreaks on average were terminated under a combined ring and reactive targeted vaccination scheme when interventions occurred after 21 days, this decreased to 82% with a delay of 90 days. This also translates to an increase in the median number of cases for these reactive vaccination approaches. Again taking the combined ring and reactive approach, we observed a small but significant increase from 31 (baseline) to 41 cases.

When we model a vaccine that prevents disease but not transmission, the proportion of terminated outbreaks remains the same as the scenario with no vaccination: 92 and 68% when the rate of introductions is low and high, respectively, for all vaccination scenarios (Table 3). The median number of cases remains low—between 1 and 3 cases—as is the case when vaccination blocks transmission (Table 4). However, at the high rate of introductions, the median case numbers between the transmission- and disease-preventing vaccines differs slightly. We see that there are fewer cases in both prophylactic scenarios when the vaccine is transmission-preventing (mass: 17 vs 20, targeted: 11 vs 21), but more in all reactive scenarios (mass: 32 vs 22, targeted: 32 vs 20, ring: 35 vs 28, ring/targeted: 31 vs 14).

Reducing the rate of introductions by one and two orders of magnitude led to an increase in the proportion of controlled outbreaks and a decrease in the median number of cases from baseline values (Tables 3 and 4). The higher introductions scenario was particularly sensitive to this change; in simulations without vaccination, the proportion of controlled outbreaks increased from 68% at baseline to 93% if the rate was reduced by two orders of magnitude, while the median number of cases per simulated outbreak decreased from 38 to 2.

## Discussion

We combined and analysed data from each of the known MVD outbreaks to characterise key epidemiological parameters and assess the potential impact of a range of vaccination strategies. We found that the reproduction number of MVD in human populations is often low but very variable, consistent with the small and mostly self-limited nature of most outbreaks. Across all outbreaks, our estimates of ${R}_{0}$ are lower than that calculated by Ajelli and Merler [33] (1.59; 95% CI 1.53–1.66). This is largely because their estimates were based on data from the peak of the Angola outbreak alone—the largest recorded outbreak—whereas ours include all of the available data up to 2021. Our estimated serial interval (9.2 days with a standard deviation of 4.4 days) is comparable to the generation time estimated by Ajelli and Merler (9 days with a standard deviation of 5.4 days) from nonhuman primates [33] and shorter than that of the Ebola Virus disease (Zaire species)—see Table 5.

**Table 5:** Median number of MVD cases per outbreak predicted during different vaccination schemes. The numbers in parentheses indicate the 95% CIs of this median, from bootstrapped sampling

**Scheme**	**No vaccination**	**Prophylactic mass**	**Prophylactic targeted**	**Reactive mass**	**Reactive targeted**	**Ring**	**Ring and reactive targeted**
**Baseline (Low/High rate of introductions)**	3 (3–3)	2 (2–2)	1 (1–1)	3 (3–3)	3 (3–3)	3 (3–3)	3 (3–3)
	36 (35–37)	17 (16–17)	11 (11–12)	32 (32–33)	32 (31–32)	35 (34–35)	31 (30–31)
**Reduced time from reactive vaccination to infective contact (Low/High rate of introductions)**	3 (3–3)	2 (2–2)	1 (1–1)	3 (3–3)	3 (3–4)	3 (3–3)	3 (3–3)
	36 (35–37)	17 (16–17)	11 (11–12)	33 (32–33)	33 (33–34)	36 (36–37)	32 (32–33)
**Lower Vaccination coverage (Low/High rate of introductions)**	3 (3–3)	3 (3–3)	2 (2–2)	3 (3–3)	3 (3–3)	3 (3–3)	3 (3–3)
	35 (33–38)	30 (28–31)	18 (17–18)	34 (33–34)	34 (33–34)	35 (35–36)	33 (32–33)
**Higher Vaccination coverage (Low/High rate of introductions)**	3 (3–3)	2 (2–2)	1 (1–1)	3 (3–3)	3 (3–3)	3 (3–3)	3 (3–3)
	36 (35–37)	16 (16–17)	11 (11–11)	32 (31–33)	32 (31–32)	34 (34–35)	30 (29–30)
**Later date of intervention (Low/High rate of introductions)**	3 (3–4)	2 (2–3)	1 (1–1)	3 (3–3)	3 (3–3)	3 (3–3)	3 (3–3)
	52 (50–55)	18 (17–18)	12 (12–12)	44 (42–46 )	43 (41–44)	47 (46–49)	41 (40–43)
**Vaccine protects against disease only (Low/High rate of introductions)**	3 (3–3)	2 (2–2)	2 (2–2)	3 (2–3)	1 (1–2)	3 (3–3)	2 (2–2)
	36 (35–37)	20 (20–21)	21 (21–22)	22 (22–23)	20 (20–21)	30 (30–31)	14 (14–15)
**Reduced introduction rate (by 1 order of magnitude)**	2 (2–2)	1 (1–1)	1 (1–1)	2 (2–2)	2 (2–2)	2 (2–2)	2 (2–2)
	6 (6–6)	3 (3–3)	2 (2–2)	5 (5–5)	5 (5–5)	5 (5–6)	5 (4–5)
**Reduced introduction rate (by 2 orders of magnitude)**	2 (2–2)	1 (1–1)	1 (1–1)	2 (2–2)	2 (2–2)	2 (2–2)	2 (2–2)
	2 (2–2)	1 (1–1)	1 (1–1)	2 (2–2)	2 (2–2)	2 (2–2)	2 (2–2)

Although our estimates of the reproduction number after interventions are generally low, this is no guarantee that all future outbreaks will be limited in size. Large future outbreaks of MVD cannot be ruled out. As the Angolan outbreak showed, for instance, a large outbreak can arise from a very limited number of introductions. Our data suggest that the time to detection of outbreaks is the main driver for outbreak size. Larger outbreaks are more likely given a long delay before implementation of interventions and/or difficulty or reluctance to adhere to these interventions. This is indeed what occurred during the two largest outbreaks (DRC in 1998–2000 and Angola in 2004–2005): in DRC, miners working illegally in a small town were continually exposed to infected bats, with transmission going undetected for months, while the Angolan outbreak occurred in a densely populated city, thus increasing person-to-person transmission. Moreover, there was a lack of trust in the authorities and so interventions such as isolation of cases took time to be properly implemented [23]. This last point hints at the many social, epidemiological and environmental factors that may likely influence outbreak size. The two aforementioned outbreaks also occurred in populations that had recently been affected by civil war [19, 23, 34], which likely resulted in fragile health systems unable to prevent or rapidly control outbreaks.

According to our mechanistic model, other factors being equal, higher introduction rates would be expected to result in a larger outbreak. However, this effect is rather small (compare result with high and low introduction rates), the timing of interventions being much more important (as is seen in the regression model). Furthermore, the MVD outbreaks have been highly heterogeneous (see Fig. 1), occurring over a very wide geographical area and over time, so it is impossible to argue that these outbreaks are otherwise equal. Finally, chance also plays a large role in determining the number of cases. In our mathematical model we can reduce this effect by undertaking a large number of simulations. However, we only have a handful of real-world outbreaks to analyse and so the ability to pick up relatively small signals amongst this stochastic noise is difficult.

To help understand what role vaccines might play in controlling future outbreaks, we developed a simple branching process model and parameterised it from our analyses of the epidemiology. As expected, vaccination generally increased the probability of outbreaks being terminated compared to no vaccination. Over the range of strategies and parameter values considered, generally similar effects were achieved, though at baseline, the prophylactic strategies as well as the combination of ring and targeted vaccination approaches were the best-performing. Exceptions included reactive targeted vaccination when the rate of spillover introductions is low. Vaccination could also be expected to reduce the median outbreak size, though this reduction is often relatively small since the median number of cases for the no vaccination scheme is already low (3 when the rate of introduction is low, 36 when high). Hence, the aim of vaccinating against MVD would be to prevent these large outbreaks from occurring.

The two prophylactic vaccination strategies, as well as a combined ring and targeted vaccination, were generally the most effective options, since they result in a high probability of termination and a low median outbreak size. However, in the case of the latter, if there are few introductions, the added effect of targeted vaccination over ring vaccination alone is negligible. Nevertheless, if a reactive scheme is indeed required, the combined approach might still be preferred since the rate of spillover introductions might be difficult to assess in real time without comprehensive sequence data.

We included a sensitivity analysis where we modelled a vaccine that works by only protecting against disease, rather than preventing transmission. Since very little transmission occurs in the absence of a vaccine, we found that this made a small difference to the median number of cases. The proportion of terminated outbreaks decreased to that of the no vaccination scenario. Higher rates of introductions also meant that a disease-preventing vaccine led to fewer cases than a vaccine that prevented transmission. The median number of cases post-intervention in the absence of vaccination was 31. Moreover, 21 of these cases were zoonotic introductions while the other 10 occurred through secondary transmission (the median post-intervention reproduction number was only 0.3, resulting in few secondary infections). A disease-preventing vaccine would work on all these 31 cases, but a transmission-preventing vaccine would only prevent the 10 secondary cases.

At baseline, we have estimated the low and high spillover rates according to the rates observed during two outbreaks assumed to be representative of a community and spillover-driven epidemic, respectively. However, in a sensitivity analysis, we also reduced these rates by one and then two orders of magnitude. This reflects the fact that the introduction rate can also be estimated across time—from 1967, when the first outbreaks were recorded—up until now. We found that the proportion of controlled outbreaks increased and the median number of cases decreased, especially under the scenario involving the higher rate of introductions (Tables 3 and 4). This is because at these much lower spillover rates, the outbreaks become predominantly community-driven.

Quantifying the number of hypothetical vaccine doses that may have been required to help control the previous outbreaks of MVD is difficult as the target population is unclear for most of these outbreaks. However, it is possible to obtain a rough idea of this for a few of the outbreaks. The DRC outbreak of 1998–2000 only affected two towns with a combined population of approximately 85,000 [21]. Had a vaccine been available the number of vaccine courses required for a targeted strategy would be 24,000, assuming 40% of the population were miners (i.e. 80% of males) and a 70% coverage rate. A mass vaccination strategy with 50% coverage, on the other hand, would require 43,000 vaccines. This outbreak does, however, constitute an extreme case where a large proportion of the population were considered high-risk individuals and thus candidates for receiving a vaccine under a targeted vaccination scheme. In comparison, the outbreak in Uganda, 2012, spanned 3 of the country’s districts and was instigated by only a single known zoonotic case. Although it is not known what fraction of the population was high risk, applying the fraction of miners and healthcare workers in the country as a whole to these districts gives a possible indication (i.e. 5600 high risk individuals amongst a total population of 730,000) [20]. Hence, 3900 vaccines would have been required under a targeted vaccination scheme with 70% coverage and 370,000 vaccines (two orders of magnitude greater) would have been required under a mass vaccination scheme with 50% coverage.

Although efforts are ongoing to develop MVD vaccines [4], the results of our study suggest that it may be difficult to carry out Phase 3 trials, since we predict that few cases will be observed in a typical outbreak, and these may well be rapidly controlled by other interventions. To counter this problem, the World Health Organization (WHO) has developed a Core Protocol approach that is designed to allow trial results to be combined across multiple outbreaks to accrue sufficient data and statistical power to assess vaccine efficacy [29, 35]. There has also been a recent paper from WHO on a core protocol for estimating Marburg virus vaccine efficacy [35]. The paper proposed that the efficacy of a potential vaccine may be estimated after a Phase 3 trial involving 150 MVD cases across multiple outbreaks at 90% power, with a 30% null and no rejection occurring after interim analyses. Simulations of our model under several plausible scenarios may be helpful for estimating the number of outbreaks required to reach these 150 cases.

A major limitation of our study is the lack of data due to the infrequency of MVD outbreaks, with most being relatively small, and in many cases, scant availability of epidemiologic data. This paucity of data and heterogeneity across and within outbreaks leads to wider credible intervals associated with ${R}_{0}$ and $E$. This heterogeneity can be seen in Fig. 1 and Table S1 from Additional file 1. There were many smaller outbreaks (7 with fewer than 3 cases) and, for these in particular, the low transmissibility before interventions may have already limited their size. Since 90% of our estimates of the post-intervention reproduction number were below 1, and all except 2 recorded MVD outbreaks have ended only weeks after interventions, it is likely that non-pharmaceutical interventions have been capable of reducing the reproduction number below 1. However, the wide credible intervals imply that we cannot be certain. If indeed the reproduction number had remained above 1 even after interventions, there would be a good case for better public health interventions, including the use of vaccination programmes.

Since the model does not take unobserved cases into account, it is possible that our estimates of the reproduction number are underestimates. However, shortly after the 2019 outbreak in Uganda, a seroprevalence study was conducted on individuals living close to caves inhabited by bats; only 1 of 433 high-risk individuals was found to have antibodies against MVD [36]. Another study conducted after the DRC outbreak found that 2 out of 121 household contacts of known MVD cases were seropositive, both of whom reported becoming ill after contact [24]. Moreover, most cases appear to result in severe illness and symptoms [16]. This suggests that there likely may not be a large number of unrecognised MVD cases, even in high-risk communities.

A lack of data on the time when different interventions were implemented (other than the very first day of the intervention response) also meant that we chose to model the intervention efficacy as a constant, rather than time-varying function. Another limitation is that, while we estimated and used a constant rate of zoonotic introductions, in reality, the rate often varies as a function of time. For example, the outbreak in the DRC appears to have been driven by seasonal introductions into miners [6]. However, due to a lack of data on zoonotic introductions into specific persons, we opted for model simplicity and chose a constant rate for each outbreak.

We chose to use a simple branching process model that does not include, for instance, depletion of the susceptible population. Depletion of susceptibles may well be an important consideration, especially under a mass vaccination strategy. Nor did we account for any possible waning of immunity post-vaccination. At present, there is a lack of data on the vaccines currently under development—we have informed this part of our analysis by making broad and simplistic assumptions. Several vaccines are currently in the pipeline [4] and vaccine parameters of our model will be updated when more information on potential vaccines become available.

## Conclusions

Our study shows that various vaccination strategies can be effective in helping to control outbreaks of MVD, with the best approach varying with the particular epidemiologic circumstances of each outbreak. Of course, many logistical and economic factors must be considered. Further studies on the economic factors involved in vaccinating against MVD will be required but are beyond the scope of this study*.* Given the rarity and generally small size of MVD outbreaks, prophylactic mass vaccination of large populations is unlikely to be feasible or warranted. However, as has been proposed for vaccination for Ebola virus, vaccination for relatively infrequent but dangerous emerging infectious diseases might be incorporated into comprehensive vaccination for numerous diseases, serving as a driver of broader health systems strengthening [37]. The rationale for this approach would be further strengthened by development of pan-filovirus vaccines, for which research is underway [38], especially if protection is long-lasting.

### Supplementary Information

**Additional file 1:** **Figure S1.** Vaccine efficacy modelled as a function of days after vaccination. **Figure S2.** Serial interval, modelled as a gamma distribution. **Figure S3.** Number of secondary marburgvirus cases as a function of A. proportion of zoonotic cases, B. days until intervention and C. year of outbreak.

## Data Availability

The code and data used in these analyses are found at: https://github.com/GeorgeYQian/Marburg_Branching_Process_Model

## Abbreviations

MVD: Marburg virus disease
DRC: Democratic Republic of Congo
${R}_{0}$: Basic reproduction number
VE: Vaccine efficacy
CI: Credible intervals
s.d.: Standard deviation
WHO: World Health Organization
