# Supplementary material for: A mathematical model of Marburg virus disease outbreaks and the potential role of vaccination in control
Source: BMC Med. 2023 Nov 14;21:439. doi: 10.1186/s12916-023-03108-x (PMC10648709; doi:10.1186/s12916-023-03108-x)
Supplement: Supplementary file 1 — Additional file 1: Figure S1. Vaccine efficacy modelled as a function of days after vaccination. Figure S2. Serial interval, modelled as a gamma distribution. Figure S3. Number of secondary marburgvirus cases as a function of A. proportion of zoonotic cases, B. days until intervention and C. year of outbreak. [file 12916_2023_3108_MOESM1_ESM.docx]

**Technical Appendix**

**Figures**


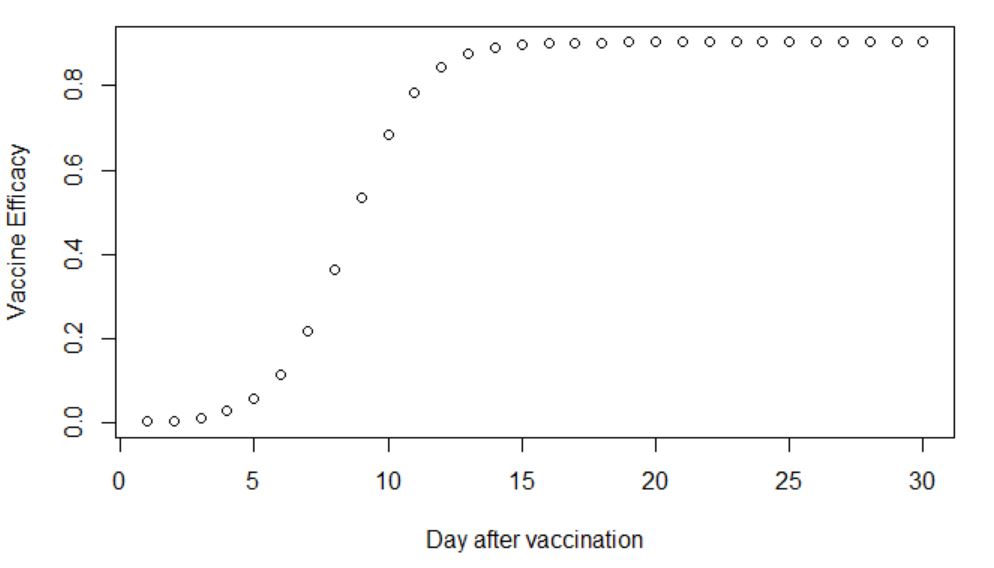


**Figure S1:** Curve showing vaccine efficacy as a logistic function of days after vaccination

**
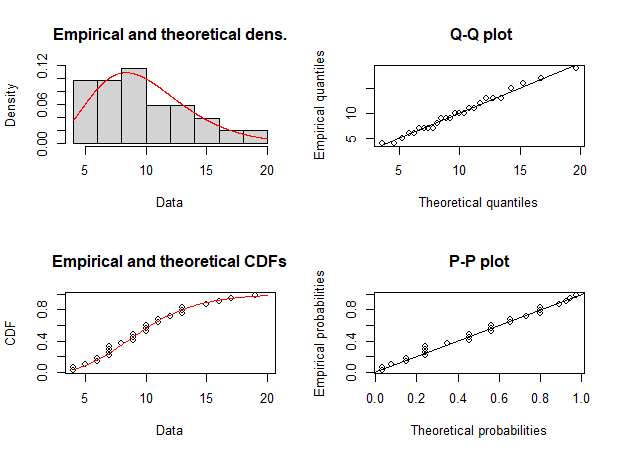
**

**Figure S2:** Serial interval, modelled as a gamma distribution


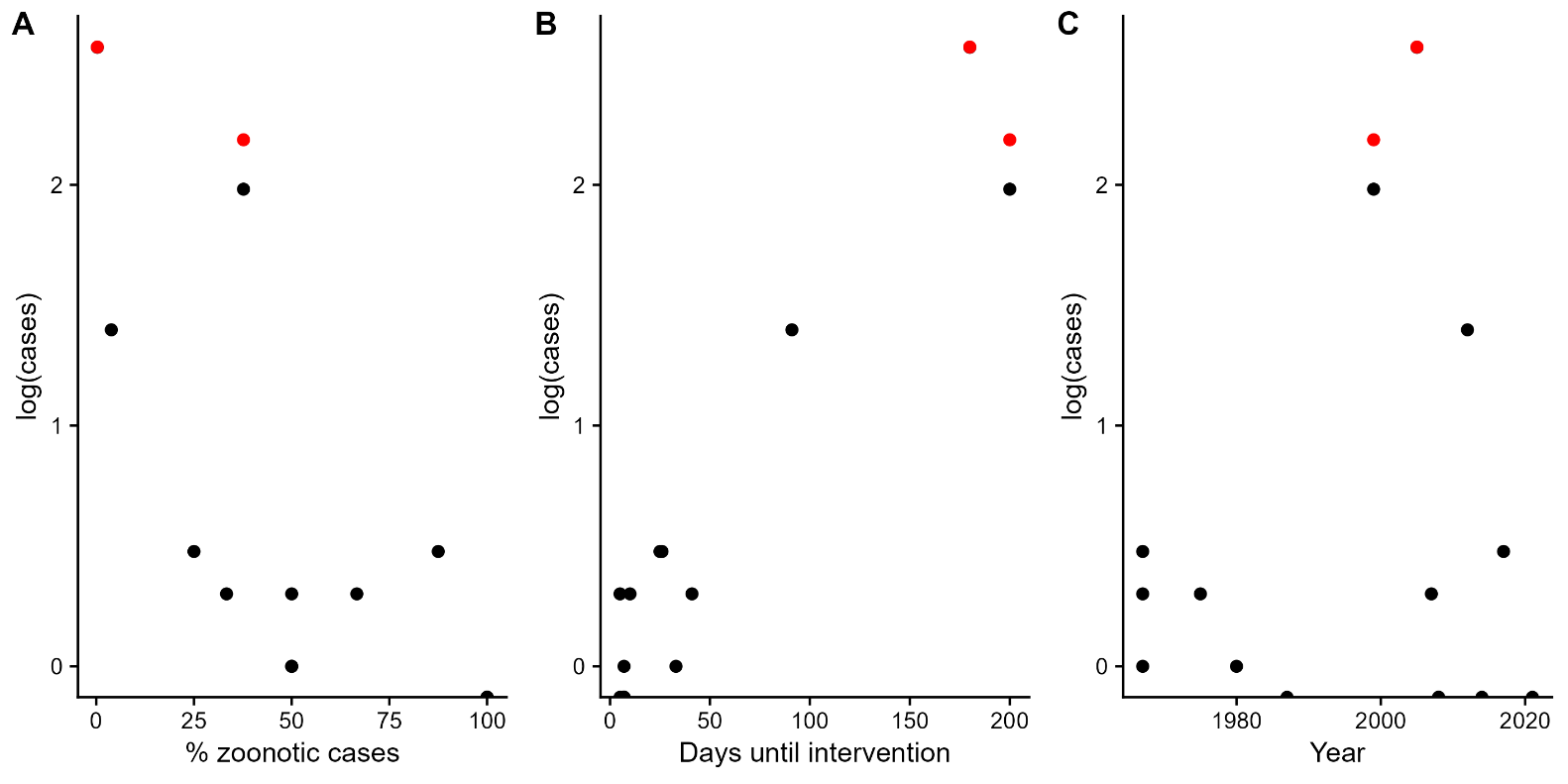


**Figure S3:** Scatterplots showing the logarithm (base 10) of the number of secondary marburgvirus cases (confirmed and probable) according to: A. proportion of zoonotic cases, B. days until intervention and C. year of outbreak. Red dots indicate outbreaks affected by civil war (namely, DRC and Angola).

**Tables**

**Table S1:** Details of previous Marburgvirus outbreaks

| **Outbreak Location** | **Year(s)** | **Probable and Confirmed Cases** | **Deaths** | **Interventions Implemented** |
| --- | --- | --- | --- | --- |
| **Marburg, Germany** | 1967 | 24 | 5 | Infected monkeys euthanised; laboratory work ended or passed to labs with better protective equipment, community surveillance |
| **Frankfurt, Germany** | 1967 | 6 | 2 | Infected monkeys euthanised; laboratory work ended or passed to labs with better protective equipment, community surveillance |
| **Belgrade, Yugoslavia** | 1967 | 2 | 0 | Infected monkeys euthanised; laboratory work ended, community surveillance |
| **Johannesburg, South Africa** | 1975 | 3 | 1 | Barrier nursing, case isolation, monitoring of contacts, community surveillance |
| **Nairobi, Kenya** | 1980 | 2 | 1 | Barrier nursing, case isolation, monitoring of contacts, community surveillance |
| **Nairobi, Kenya** | 1987 | 1 | 1 | Barrier nursing, case isolation, monitoring of contacts, community surveillance |
| **Durba and Watsa, Democratic Republic of Congo** | 1998-2000 | 154 | 128 | Deployment of international medical/logistics team to affected regions, isolation unit installed, PPE supplies brought in, barrier nursing, monitoring of contacts, community surveillance, prohibiting entry into bat-infested mine |
| **Uige, Angola** | 2004-2005 | 374 | 329 | Deployment of local and international medical/logistics team to affected regions, isolation unit installed, PPE supplies brought in, barrier nursing, monitoring of contacts, community surveillance, fatalities removed by fully-protected staff, campaign to foster awareness of the importance of interventions amongst locals |
| **Uganda** | 2007 | 4 | 2 | Case isolation, barrier nursing, monitoring of contacts, community surveillance, prohibiting entry into bat-infested mine, campaign to foster awareness of dangers of bats and the mine amongst locals |
| **USA (via Uganda)** | 2008 | 1 | 0 | Barrier nursing, case isolation, monitoring of contacts (across continents), community surveillance, |
| **Netherlands (via Uganda)** | 2008 | 1 | 1 | Barrier nursing, case isolation, monitoring of contacts (across continents), community surveillance |
| **Uganda** | 2012 | 26 | 15 | Deployment of local and international medical/logistics team to affected regions, medical centres already supplied with appropriate PPE and staff had received training on handling MVD cases due to previous outbreaks, case isolation, barrier nursing, monitoring of contacts, community surveillance, fatalities removed by fully-protected staff |
| **Uganda** | 2014 | 1 | 1 | Deployment of local and international medical/logistics team to affected regions, medical centres already supplied with appropriate PPE and staff had received training on handling MVD cases due to previous outbreaks, case isolation, barrier nursing, monitoring of contacts, community surveillance, fatality removed by fully-protected staff |
| **Uganda** | 2017 | 4 | 1 | Deployment of district and national medical/logistics team to affected regions, medical centres already supplied with appropriate PPE and staff had received training on handling MVD cases due to previous outbreaks, case isolation, barrier nursing, monitoring of contacts, community surveillance, fatality removed by fully-protected staff |
| **Guinea** | 2021 | 1 | 1 | Deployment of district and national medical/logistics team to affected regions, case isolation, training of staff at health clinics, barrier nursing, monitoring of contacts, community surveillance |

**Table A2:** Akaike Information Criteria (AIC) associated with four model fits of the serial intervals associated with Marburg Virus Disease. The resultant fit for the Gamma Distribution is shown in Figure A2.

| **Distribution** | **AIC** |
| --- | --- |
| **Gamma** | 146 |
| **Negative binomial** | 147 |
| **Poisson** | 147 |
| **Logistic** | 150 |
